# Technical Feasibility of Microwave Ablation in Pancreatic Tumors: A Scoping Review of Procedural Efficacy and Safety

**DOI:** 10.3390/cancers17071197

**Published:** 2025-03-31

**Authors:** Daniela Tabacelia, Carlos Robles-Medranda, Artsiom Klimko, Stephen P. Pereira, Peter Vilmann, Rogier P. Voermans, Adrian Săftoiu, Cristian George Tieranu, Cezar Stroescu

**Affiliations:** 1 Faculty of Medicine, Carol Davila University of Medicine and Pharmacy, 010017 Bucharest, Romania; daniela.tabacelia@drd.umfcd.ro (D.T.); adriansaftoiu@gmail.com (A.S.); tieranucristian@gmail.com (C.G.T.); 2 Elias Emergency University Hospital, 010017, Bucharest, Romania; 3 Ecuadorian Institute of Digestive Diseases, Guayaquil 090101, Ecuador; carlosoakm@yahoo.es; 4 Balgrist University Hospital, 8000 Zurich, Switzerland; 5 Institute for Liver and Digestive Health, University College London, London NW3 2QG, UK; stephen.pereira@ucl.ac.uk; 6 Gastro Unit, Division of Endoscopy, Herlev and Gentofte Hospital, 2730 Herlev, Denmark; peter.vilmann@gmail.com; 7 Department of Clinical Medicine, University of Copenhagen, 1050 Copenhagen, Denmark; 8 Department of Gastroenterology & Hepatology, Amsterdam Gastroenterology and Metabolism, Amsterdam UMC, University of Amsterdam, Meibergdreef 9, 1105 AZ Amsterdam, The Netherlands; r.p.voermans@amsterdamumc.nl; 9 Surgery Department, Fundeni Clinical Institute, 010017 Bucharest, Romania; cezar.stroescu@gmail.com

**Keywords:** microwave ablation, pancreatic cancer, locally advanced pancreatic cancer, pancreatic neuroendocrine tumors, renal cell carcinoma, minimally invasive therapy

## Abstract

Background/Objectives: Pancreatic cancer remains one of the most aggressive and lethal malignancies, with limited effective treatment options for advanced stages. Microwave Ablation (MWA) has emerged as a minimally invasive therapeutic modality, offering potential benefits in tumor control. This review aims to critically assess the safety and efficacy of MWA in the treatment of pancreatic cancer, focusing on its application in various pancreatic lesions. Methods: We systematically reviewed studies published between 2010 and 2023 that evaluated the use of MWA in pancreatic tumors, including locally advanced pancreatic cancer (LAPC), pancreatic neuroendocrine tumors (PNETs), and pancreatic metastases from renal cell carcinoma (RCC). Due to limited data on survival rates and long-term outcomes, our analysis concentrated primarily on the technical aspects and immediate procedural outcomes of MWA. Results: MWA was technically feasible in all cases. The overall complication rate was approximately 16.7% (nine patients), with higher incidences in tumors located in the pancreatic head. Reported complications included pancreatitis and pseudocyst formation. Procedural parameters varied, with applied energy ranging from 20 to 80 watts and ablation times between 2 to 15 min, depending on the microwave generator type and approach (percutaneous, intraoperative or endoscopic). All cases demonstrated effective necrosis of the target tissue, and several studies reported notable tumor size reductions, averaging up to 70%. Conclusions: MWA shows promise as a therapeutic option for pancreatic cancer, achieving high technical success rates and significant tumor reductions. However, the procedure is associated with a moderate complication rate, particularly in tumors located in the pancreatic head.

## Simple Summary

Pancreatic tumors are diagnosed with increasing frequency, thus comprising significant routine conditions in clinical practice. In addition to the current approaches, which include surgery, chemotherapy and radiation treatment, local ablative therapies provide the potential of an additional therapeutic tool. This review collected the existing literature regarding microwave ablation for pancreatic tumors and assessed its efficacy and safety. MWA was technically feasible in all cases with effective necrosis of the target tissue, and some, with notable tumor size reductions, averaging up to 70%. Considering safety, the overall complication rate was approximately 16.7%, with higher incidences in tumors located in the pancreatic head and severity ranging from mild to serious. MWA pancreatic tumor ablation seems to be an acceptable and safe procedure, with promising results in appropriately selected patients.

## 1. Introduction

Pancreatic ductal adenocarcinoma (PDAC) is among the most aggressive malignancies, characterized by late diagnosis, rapid progression, and poor prognosis [1,2]. It is the fourth leading cause of cancer-related deaths worldwide, with an estimated 495,000 new cases and 466,000 deaths annually as of 2020 [3]. The five-year survival rate according to Cancer statistics remains approximately 12%, primarily due to the advanced stage at diagnosis and limited effective therapeutic options [4]. Surgical resection offers the best chance for long-term survival; however, only about 20% of patients present with resectable disease [5]. For patients with unresectable or locally advanced pancreatic cancer (LAPC), the standard of care includes chemotherapy and chemoradiation, which have a largely palliative role and significant toxicities [5,6].

Minimally invasive local ablative techniques have gained prominence as alternative or adjunct therapeutic options for managing pancreatic lesions, especially in patients who are high-risk surgical candidates or have unresectable tumors [7,8]. Among these techniques, radiofrequency and microwave ablation, irreversible electroporation (IRE) and high intensity focused ultrasound (HIFU) have been performed in different studies with satisfactory results concerning the local treatment of pancreatic tumors. In comparative studies for liver tumors, microwave ablation (MWA) has emerged as a promising modality due to its ability to achieve higher intra-tumoral temperatures rapidly, providing larger and more uniform ablation zones compared to other thermal ablation methods like radiofrequency ablation (RFA) [9,10]. Additionally, MWA is less susceptible to the ‘heat sink’ effect caused by nearby blood vessels, which can impede the efficacy of thermal ablation by dissipating heat away from the target area [11,12]. Unlike radiofrequency ablation (RFA), some MWA devices allow multiple ablation antennas insertion at once. No current RFA systems can drive multiple electrically independent electrodes simultaneously [13]. This capability may shorten procedure times and increase efficiency, particularly in treating multiple or large tumors (>5 cm).

Microwave ablation relies on oscillation of water molecules to generate frictional heating, tissue necrosis and apoptosis. In addition to that, ablative therapies may induce immunogenic cell death, with subsequent release of tumor antigens and damage-associated molecular patterns (DAMPs) stimulating an antitumor immune response [14,15]. This raises the possibility of combining MWA with immunotherapies to improve outcomes in PDAC, a tumor type known for its immunosuppressive microenvironment [16,17]. Beyond its potential as a standalone treatment, MWA can also act as a bridge to other therapies, such as chemotherapy, radiation therapy, or surgery by acting as a cytoreductive procedure. By reducing tumor size or alleviating symptoms, MWA may improve a patient’s overall condition, making them more eligible for additional treatments and potentially improving their outcomes [18,19]. Within this context, we aim to evaluate the clinical performance of MWA as a local therapy of pancreatic tumors, focusing on safety and efficacy of this treatment modality.

## 2. Materials and Methods

We conducted a systematic literature search of PubMed, MEDLINE, and Embase databases from January 2010 to October 2024 using keywords such as “microwave ablation”, “pancreatic cancer”, “locally advanced pancreatic cancer”, “immunomodulation”, and “thermal ablation”. The inclusion criteria were studies reporting clinical outcomes of MWA in pancreatic tumors, providing data on procedural details, efficacy, safety, and immunomodulatory effects. Additionally, our review includes a cohort of 10 patients treated with MWA for pancreatic cancer, previously presented at the 8th EUS-ENDO Conference in Marseille, France [20]. Exclusion criteria included studies without full-text availability, non-English language articles, case reports with fewer than three patients, and studies focusing on animal models or in vitro experiments. The MWA devices used were represented by the first generation MWA system and the Emprint Thermosphere ablation devices from Medtronic that use frequencies between 915 MHz, and 2.45 GHz, respectively coupled to 14G antennas with a 3 cm active tip. For the EUS approach, a Croma generator from Creo Medical attached to a 19G antenna with an active point in the tip of the needle was used.

Data was independently extracted by two reviewers using a standardized form, collecting information on study design, patient demographics, tumor characteristics, procedural details, and outcomes. Ablation time refers to the duration of microwave energy application during the procedure. Safety outcomes involved recording complications such as bleeding or infection, with complications categorized according to the Clavien-Dindo classification [21].

Given the heterogeneity in patient characteristics, tumor types, and treatment protocols across the studies, the results were synthesized qualitatively rather than a meta-analysis. One study was excluded due to the unavailability of the full-text article [9]. Reference lists of included studies were also reviewed for additional relevant articles. Data extracted from the studies were analyzed using descriptive statistics. Results were organized into tables summarizing patient characteristics, ablation time, safety outcomes, efficacy, and follow-up data. For studies providing individual patient information, each patient was assigned an identification number to link data across tables.

## 3. Results

Our literature review included six studies, with a total of 54 patients (34 males and 21 females) and the findings are summarized in Table 1. The tumor types consisted of 33 cases of pancreatic ductal adenocarcinoma (PDAC), 18 cases of neuroendocrine tumors (NETs), and 4 cases of pancreatic metastases from renal cancer (RMTs). The procedural success rate of MWA was high across all studies, with a success rate close to 100% in each study. In a cohort of 10 patients treated by endoscopic guidance, which also included cases of PDAC, NETs, and RMTs, the success rate was 100% and complications were minimal, with only two acute issues (minimal hemosuccus pancreaticus and mild gastric oozing not requiring blood transfusion) occurring in patients with NETs [20].

Robles-Medranda et al. reported a successful MWA procedure in a single female patient with a neuroendocrine tumor located in the neck of the pancreas, with no complications noted [22]. Egorov et al. included eight patients with neuroendocrine tumors primarily located in the head of the pancreas [19]. This study also reported a 100% procedural success rate, although late complications were observed in three cases, including pancreatic head pseudocysts and pancreatic fistula.

Ierardi et al. evaluated five patients with PDAC, all of whom had successful MWA procedures reporting peripancreatic fluid collection in one case [23]. D’Amico et al. presented a single case of a male patient with an intraductal papillary-mucinous neoplasm in the head of the pancreas [24]. The procedure was successful without any complications. Vogl et al. included 20 patients with PDAC, achieving high procedural success but reporting severe local pain [15]. Carrafiello et al. involved ten patients with PDAC in the head of the pancreas [18]. While the procedural success rate was high, complications were reported in three patients including pancreatitis, pseudocyst formation, a pseudoaneurysm of the gastroduodenal artery that required embolization [18].

In Table 2, we provide a more granular breakdown of the encountered procedural complications and their severity. In the cohort where the EUS approach was used, there was one case of hemosuccus pancreaticus and one patient with a mild gastric bleed; both spontaneously subsided (Clavien-Dindo Grade I). Other studies reported three patients with late complications, including two cases of pancreatic head pseudocyst (one Clavien-Dindo Grade 3a and one Grade I) and one case of pancreatic fistula (Clavien-Dindo Grade 3a) [19]. Additionally, severe local pain (Clavien-Dindo Grade II) was reported in one patient [15]. Three other complications included one case of pancreatitis (Clavien-Dindo Grade I), one case of pancreatitis leading to pseudocyst formation (Clavien-Dindo Grade 3a), and one case of pseudoaneurysm of the gastroduodenal artery (Clavien-Dindo Grade 4) [18].

The overall complication rate across the studies was approximately 16.7%, with severity ranging from mild to serious. The most severe complication observed was a pseudoaneurysm of the gastroduodenal artery, classified as Clavien-Dindo Grade 4, requiring emerging endovascular therapy by the interventional radiologist [18]. The occurrence of complications such as pancreatic head pseudocyst and postoperative pancreatic fistula, both necessitating additional minimal invasive interventions (Grade 3a), emphasizes the importance of vigilant post-procedural monitoring and management.

MWA procedures were performed under ultrasound guidance, endoscopic ultrasound (EUS) or computed tomography (CT). The frequency of the microwave generators used varied between 0.915 GHz, 2.45 GHz, and 5.8 GHz. The energy applied and the duration of ablation differed across studies, typically ranging from 20 to 100 watts and 2 to 10 min, respectively. When reported, the ablation diameter ranged from 2 to 3 cm. These variations in procedural parameters suggest a need for standardization and optimization of MWA techniques in future research. Beyond its safety profile, MWA demonstrated effectiveness in reducing tumor size, with some studies noting an average tumor reduction of 70% [19,23]. These results suggest that MWA could be particularly beneficial for patients who are not suitable candidates for surgical resection due to factors such as tumor location, comorbidities, or other considerations.

Carrafiello et al. evaluated the efficacy of MWA in unresectable pancreatic head adenocarcinomas, observing a 30% rate of MWA-related morbidity over an average 9.2-month follow-up [18]. They found pancreatitis in two patients and a gastroduodenal artery pseudoaneurysm in one patient. These results point to a relatively high technical success rate of MWA, with manageable complications, further supporting its use in managing pancreatic cancer. The study by Ierardi et al. reported a 100% technical success rate for MWA in treating unresectable pancreatic cancer [23]. They also noted improvements in the quality of life for patients, which, along with minimal complications, emphasizes MWA’s potential for improving patient outcomes in pancreatic cancer treatment. Egorov et al. presented the first experience of using MWA in the management of pancreatic neuroendocrine tumors [19]. They highlighted MWA’s advantages, such as shorter procedure times compared to RFA, wider active heating zone, and higher temperatures in the target zone. However, the study was more focused on the technical aspects of MWA and did not provide detailed statistics on complications or pain management. In a case report assessing a new high-power MWA device, Robles-Medranda et al. reported a 100% technical success rate and an overall effectiveness of 92.8% [22]. The paper provides an insight into the advancements in MWA technology, further solidifying its role in the treatment of pancreatic tumors.

Analysis of procedural parameters reflects relative heterogeneity in the settings used (Table 3). The approaches and guidance techniques varied, including intraoperative and percutaneous ultrasound (US), endoscopic ultrasound (EUS) and percutaneous computed tomography (CT) guidance. The approaches and guidance techniques, which are the two variables considered, appear to be independent of tumor size and location. The observed variation in tumor sizes, generator frequencies, applied energy, and ablation times highlights the need for standardized procedural parameters to optimize outcomes and minimize complications. The differences in approach and guidance techniques also suggest that the choice of method may depend on tumor location and patient-specific factors.

## 4. Discussion

Pancreatic cancer is characterized by a dismal prognosis, largely due to late presentation and the aggressive nature of the disease, which limits the effectiveness of current therapeutic options such as surgery and chemoradiation [8]. Our review highlights the technical feasibility and safety of MWA in the local treatment of pancreatic cancer, with studies demonstrating high procedural success rates and acceptable complication profiles [12,15,18,23].

MWA has been well studied in the context of hepatic, pulmonary, and renal cancer, with large cohorts of patients and multi-year follow-up providing high-powered evidence of the techniques’ safety, efficacy, and specific advantages and disadvantages compared to other ablation techniques.

Notably, Vogl et al. reported a 100% technical success rate in their series of 20 patients undergoing percutaneous MWA for LAPC, with minor complications occurring in 9.1% of cases [15]. This suggests that a percutaneous approach, guided by imaging modalities such as CT or US, can effectively deliver MWA without the need for surgical intervention, potentially reducing patient morbidity and improving quality of life in the palliative setting [15]. Similarly, Carrafiello et al. demonstrated that both percutaneous and laparoscopically MWA are feasible, with a technical success rate of 100% in their cohort [18].

Unlike RFA, MWA harnesses high-frequency electromagnetic waves to generate frictional heating through the oscillation of charged ions and polar molecules (e.g., water)—this ultimately causes hyperthermia, cellular injury and coagulative necrosis of the ablated zone. As such, tissue charring and its resulting electrical impedance do not affect MWA efficacy. Therefore, MWA is less susceptible to the vascular heat sink effect, rapidly producing large, uniform ablation areas with high intra-tumoral temperatures.

EUS guidance is now the gold standard for diagnostic and interventional procedures in pancreatic cancer due to the pancreas’ complex anatomical location and its proximity to major blood vessels and vital organs. The pancreas is situated deep within the abdomen and is surrounded by various structures, making tumors challenging to visualize and access. EUS provides real-time imaging, which is essential for precise needle placement during and for ensuring thorough tumor coverage. This level of precision is especially important in pancreatic cancer to minimize the risk of damaging nearby organs and structures. Until now, numerous studies have evaluated the use of EUS RFA in the management of pancreatic lesions including cystic tumors, PNTEs, PDAC and RMTs with very satisfactory results [25,26]. The EUS RFA system also consists of a generator that uses moderate-frequency alternative current in the range of 400–500 kHz, a 19G needle electrode and a cooling pump that internally is cooling the electrode needle with saline to prevent charring on the electrode surface [26]. The RFA system also contains grounding pads which increases the risk of skin burn compared to MWA [8,25,26].

When the EUS approach was used, the MWA procedure utilized a 19G needle antenna (MicroBlate fine, Creo Medical, Chepstow, Wales, UK) to deliver a frequency of 5.8 GHz continuously for 2 min and multiple applications [20,22]. This approach and specific frequency highlight the technical advancements in MWA, particularly in terms of frequency range and precision in targeting lesions. The use of a 5.8 GHz frequency in MWA is notable as it’s significantly higher than the commonly used frequencies in most MWA procedures. Higher frequencies than RFA can provide better control and precision in ablation, potentially leading to more effective treatment outcomes. The use of a 19G needle antenna also suggests a focus on minimizing invasiveness while ensuring effective energy delivery to the targeted area.

CT scans are used post-procedurally to check for the success of the tumor ablation and to detect any procedural early or late adverse effects [15,18]. US guidance is particularly advantageous when dealing with tumors located in organs that are easily visualized by ultrasound, such as the liver or kidney.

Percutaneous technique is different from EUS because of the risk of the procedure, the precision of targeting the lesion and especially because both deliver different amounts of energy to the lesion. This review underlines the differences and at the same time the progress in the way pancreatic tumors are approached. EUS guidance is preferred due to its real-time imaging capabilities, which allow for precise localization of therapy. It is less complex and less invasive than CT. The real-time nature of ultrasound allows for dynamic adjustment of the needle position during the procedure.

On an analysis on 176 patients with stage III and IV pancreatic cancer that have been treated with chemotherapy and HIFU, compared with a group treated with chemotherapy alone (89 patients), the median survival time was significantly longer for the HIFU group (372 vs. 220 days (12.2 vs. 7.2 months)) [27]. The advantages of HIFU are the fact that the procedure can be performed without sedation, and it is a non-thermal non-invasive technique. However, the rate of adverse events was 2.8%, and included events similar to those mentioned in our review (two pseudocysts, one mild pancreatitis, one skin burn, and one gastric ulcer, all classified as mild adverse events) [27]. The limitation of HIFU is the fact that it cannot treat lesions further than 20 mm from the abdominal wall. MWA does not have this limitation and more, the EUS approach can target any lesion located in the pancreas. Another local non-thermal ablative technique with successful results on local treatment of pancreatic tumors with a percutaneous approach is IRE. The PANFIRE and PANFIRE—2 studies included 50 participants total with locally advanced pancreatic cancer, with a median OS from IRE of 17 months, 14 minor and 21 major complications in 29 patients (58% complication rate) and 2 deaths procedure related at 90 days [28,29].

It is well known that all ablative therapies activate the immune system through their local aggression and, moreover, they have a so-called abscopal effect, in which the treatment of the primary tumor exerts an antitumor effect on distant metastases [26]. Further studies are needed to better define the potential of local MWA in combination with chemotherapy and immunotherapy in the treatment of “immunologically cold” pancreatic tumors.

Our literature review has several limitations—firstly, all studies were retrospective, which are prone to selection bias and other confounding factors. The relatively small sample sizes in these studies also limit the generalizability of the results. Long-term outcomes, including recurrence rates and overall survival, were not consistently reported, making it difficult to draw definitive conclusions about the lasting impact of MWA in pancreatic cancer treatment.

## 5. Conclusions

MWA has demonstrated potential as a safe and effective treatment option for various types of pancreatic cancer, particularly in patients with unresectable tumors or those not eligible for surgery due to comorbidities or other factors. Further well-designed studies are required to better define the benefits of local MWA in the multidisciplinary treatment of pancreatic tumors.

## Figures and Tables

**Table 1:** Overview of included studies.

Author and Year	Patient ID	Sex	Age	Tumor Type	Tumor Location	Approach	Tumor Size (pre-op), mm
Tabacelia et al., 2023 [20]	10 total					EUS	
	1	Female	48	PDAC	Body		35 × 32
	2	Female	69	PDAC	Neck		40 × 41
	3	Female	74	PDAC	Tail		32 × 22
	4	Female	72	pNET	Neck		35 × 32
	5	Male	47	pNET	Body		40 × 36
	6	Male	64	pNET	Head		25 × 15
	7	Male	41	pNET	Body		24 × 15
	8	Male	68	RMTs	Body		17
	9	Male	76	RMTs	Tail		16
	10	Male	77	RMTs	Body and tail (2 lesions)		20 × 8
Robles-Medranda et al., 2022 [22]	1 total	Female	72	pNET	Neck	EUS	35 × 32
Egorov et al., 2019 [19]	7 total					Percutaneous, US	
	12	Male	81	pNET	Head		21 × 10
	13	Female	87	pNET	Head		12 × 10
	14	Female	75	pNET	Head		16 × 14
	15	Female	78	pNET	Head and body		22 × 17
	16	Male	37	pNET	Head		19 × 15
	17	Female	46	pNET	Head		15 × 14
	18	Female	55	pNET	Head		17 × 11
Ierardi et al., 2018 [23]	5 total					Percutaneous, US	
	19	Male	76	PDAC	NA		25
	20	Female	80	PDAC	NA		30
	21	Male	70	PDAC	NA		32
	22	Male	74	PDAC	NA		25
	23	Male	69	PDAC	NA		27
D’Amico et al., 2018 [24]	1 total					Percutaneous, US	
	24	Male	70	IPMN	Head		43
Vogl et al., 2018 [15]	20 total					Percutaneous, CT	
	NA	13 Male, 7 Female	60	PDAC	NA		30 (26–35)
Carrafiello et al., 2013 [18]	10 total					Percutaneous, CT	
	NA	6 Male; 4 female	66	PDAC	Head		32 (20–43)

PDAC—pancreatic ductal adenocarcinoma, pNET—pancreatic neuroendocrine tumor, RMTs—pancreatic renal metastasis, IPMN—intraductal papillary-mucinous neoplasm, EUS—endoscopic ultrasound, US—ultrasound, CT—computed tomography.

**Table 2:** List of complications associated with MWA.

Author and Year	Patient ID	Follow-Up Time (Months)	Complication	Clavien-Dindo Grade
Tabacelia et al., 2023 [20]	6	12	Hemosuccus pancreaticus	1
	7	12	Mild gastric bleed	1
Egorov et al., 2019 [19]	14	11	Pancreatic head pseudocyst	3a
	17	38	Pancreatic head pseudocyst	1
	18	33	Postoperative pancreatic fistula grade B	3a
Vogl et al., 2018 [15]	NA	3	Severe local pain	2
Carrafiello et al., 2013 [18]	NA	12	Pancreatitis	1
	NA	1	Pancreatitis leading to pseudocyst	3a
	NA	1	Pseudoaneurysm of gastroduodenal artery	4
Ierardi et al., 2018 [23]	NA	12	Peripancreatic fluid collection	1

**Table 3:** Summary of procedural parameters of MVA ablation in pancreatic cancer.

Author and Year	Patients	Approach and Guidance	Approximate Preoperative Tumor Size (mm)	MV Generator Frequency	Applied Energy	Ablation Time (min)
Tabacelia et al., 2023 [20]	10	EUS	28.6 × 26.1	5.8 GHz	20 W	2
Robles-Medranda et al., 2022 [22]	1/1	EUS	35 × 32	5.8 GHz	NA	2
Egorov et al., 2019 [19]	7	Percutaneous, US	16.1 × 14.3	NA	NA	NA
Ierardi et al., 2018 [23]	5	Percutaneous, US	27.8	2.45 GHz	100 W	2.3–3.0
D’Amico et al., 2018 [24]	1	Percutaneous, US	43	2.45 GHz	20 W	2.0
Vogl et al., 2018 [15]	20	Percutaneous, CT	30	2.45 GHz	5–100 W	2.6
Carrafiello et al., 2013 [18]	10	Percutaneous, CT	31.67	0.915 GHz	45 W	10
